# Systemic Oxidative and Nitrosative Stress in Benign Prostatic Hyperplasia

**DOI:** 10.3390/antiox15040488

**Published:** 2026-04-14

**Authors:** Marek Biesiadecki, Sabina Galiniak, Krzysztof Balawender, Julia Połeć, Mateusz Mołoń

**Affiliations:** 1 Faculty of Medicine, University of Rzeszów, al. Tadeusza Rejtana 16C, 35-959 Rzeszów, Poland; mbiesiadecki@ur.edu.pl (M.B.); kbalawender@ur.edu.pl (K.B.); jp131108@stud.ur.edu.pl (J.P.); 2 Faculty of Biology, Natural Protection, and Sustainable Development, University of Rzeszów, al. Tadeusza Rejtana 16C, 35-959 Rzeszów, Poland

**Keywords:** benign prostatic hyperplasia, oxidative stress, nitrosative stress, lipid peroxidation, kynurenine pathway

## Abstract

Benign prostatic hyperplasia (BPH) is an age-related disorder increasingly linked to chronic inflammation and redox imbalance, yet its systemic oxidative and nitrosative profile remains insufficiently characterized. In this cross-sectional study, fasting serum samples were collected from 47 men with clinically confirmed BPH scheduled for transurethral resection of the prostate and 40 healthy controls. We assessed antioxidant status (thiols, total antioxidant capacity), lipid peroxidation (malondialdehyde, 4-hydroxynonenal), protein nitration (3-nitrotyrosine), glycoxidation markers (Amadori products, advanced glycation end products (AGE)-associated fluorescence), and tryptophan metabolism indices (tryptophan, kynurenine, N′-formylkynurenine). Compared with controls, BPH patients showed significantly lower antioxidant capacity and thiol levels, together with increased lipid peroxidation and protein nitration. AGE-associated fluorescence was modestly elevated, whereas Amadori products and advanced oxidation protein products did not differ significantly. Tryptophan metabolism was markedly altered, with lower tryptophan and higher kynurenine and N′-formylkynurenine, indicating activation of the kynurenine pathway. After false discovery rate correction, most redox biomarkers remained significant. Multivariable logistic regression confirmed independent associations of lipid peroxidation, nitrosative stress, and kynurenine pathway activation with BPH after adjustment for age and metabolic parameters. These findings support a role for systemic oxidative and inflammatory mechanisms in BPH pathophysiology, although confirmation in age-matched and longitudinal studies is needed.

## 1. Introduction

Benign prostatic hyperplasia (BPH) is one of the most prevalent urological disorders in aging men and represents a major cause of lower urinary tract symptoms (LUTS), negatively affecting quality of life and increasing healthcare burden worldwide [1,2]. Between 1990 and 2021, the global prevalence of BPH increased by 122%, reaching more than 112 million affected individuals worldwide [3]. Although BPH has long been considered a hormone-dependent condition, accumulating evidence indicates that its pathogenesis is multifactorial and involves complex interactions between aging, chronic low-grade inflammation, metabolic alterations, and cellular oxidative stress [4,5,6].

Oxidative stress (OS), defined as an imbalance between the production of reactive oxygen species (ROS) and antioxidant defense mechanisms, has emerged as a key contributor to age-related tissue remodeling and chronic non-malignant disorders [7,8]. In the prostate, excessive generation of ROS may promote stromal and epithelial cell proliferation, extracellular matrix remodeling, and microvascular alterations, thereby facilitating prostate enlargement and functional obstruction [9]. Experimental and clinical studies indicate that oxidative stress can amplify pro-inflammatory signaling pathways, leading to the persistence of a chronic inflammatory microenvironment and further sustaining the progression of BPH [5,10].

Previous clinical investigations addressing oxidative stress in BPH have primarily focused on classical biomarkers, such as malondialdehyde (MDA) [11], total antioxidant capacity (TAC) [12], and selected antioxidant enzymes [11,13,14]. While these studies consistently indicate enhanced oxidative burden in patients with BPH compared with healthy individuals, the biochemical characterization of oxidative damage in this condition remains incomplete. In particular, limited attention has been paid to oxidative modifications of proteins and amino acids, as well as to non-enzymatic glycation processes that are closely linked to aging and metabolic homeostasis.

Beyond classical lipid peroxidation markers such as MDA, secondary aldehydic products of lipid oxidation, including 4-hydroxy-2-nonenal (4-HNE), represent biologically active mediators capable of modifying proteins, lipids, and nucleic acids, thereby amplifying oxidative and inflammatory signaling. In parallel, nitrosative stress-related protein modifications, particularly the formation of 3-nitrotyrosine (3-NT), reflect peroxynitrite-mediated protein damage and serve as stable indicators of chronic redox imbalance [15]. Although both 4-HNE and 3-NT have been implicated in prostate carcinogenesis [16,17], their systemic relevance in BPH remains insufficiently characterized.

Advanced glycation end products (AGEs) and early glycation intermediates, including Amadori products, accumulate progressively with age and oxidative stress, yet the involvement of glycation-associated oxidative damage in BPH has not been comprehensively investigated [18,19].

In parallel, the metabolism of aromatic amino acids, particularly tryptophan, has gained increasing attention in the context of oxidative and inflammatory stress [20]. Oxidative degradation products of tryptophan, such as kynurenine and N′-formylkynurenine, as well as protein cross-linking products like dityrosine, serve as sensitive indicators of oxidative protein damage [21]. Alterations in the tryptophan-kynurenine pathway have been implicated in immune modulation, cellular redox balance, and age-related pathologies [22]; however, their potential contribution to BPH-associated oxidative stress remains largely unexplored.

Excessive production of reactive oxygen and nitrogen species can promote chronic low-grade inflammation, contribute to stromal and epithelial alterations, induce extracellular matrix remodeling, and impair local microvascular homeostasis [23,24]. Together, these processes may favor prostatic enlargement and the progression of LUTS. 

Given these gaps in current knowledge, a more comprehensive biochemical assessment of oxidative stress in BPH is warranted. Therefore, the aim of the present study was to evaluate systemic oxidative stress and antioxidant status in patients with BPH compared with healthy controls by analyzing a broad panel of oxidative stress markers. This panel included lipid peroxidation products, total antioxidant capacity assessed by complementary methods, markers of protein oxidation, thiol group content, early glycation products, advanced glycation end products, and selected fluorescent markers of oxidative amino acid modification related to the tryptophan-kynurenine pathway. By integrating multiple biochemical pathways, this study seeks to provide a more detailed insight into the oxidative and glycoxidative milieu associated with BPH. To our knowledge, this is the first study to simultaneously integrate lipid peroxidation, nitrosative stress, glycoxidation, and tryptophan metabolism markers in a well-characterized BPH cohort.

## 2. Materials and Methods

### 2.1. Ethical Approval

The study protocol received approval from the Bioethics Committee of the University of Rzeszów (decision numbers: 2022/017, date: 9 February 2022; 2022/037, date: 6 April 2022; and 2022/090, date: 9 November 2022). All research activities were conducted in full compliance with the ethical principles of the Declaration of Helsinki. Each individual provided written informed consent before taking part in the study. All data were anonymized prior to analysis.

### 2.2. Study Group

**Figure 1:**
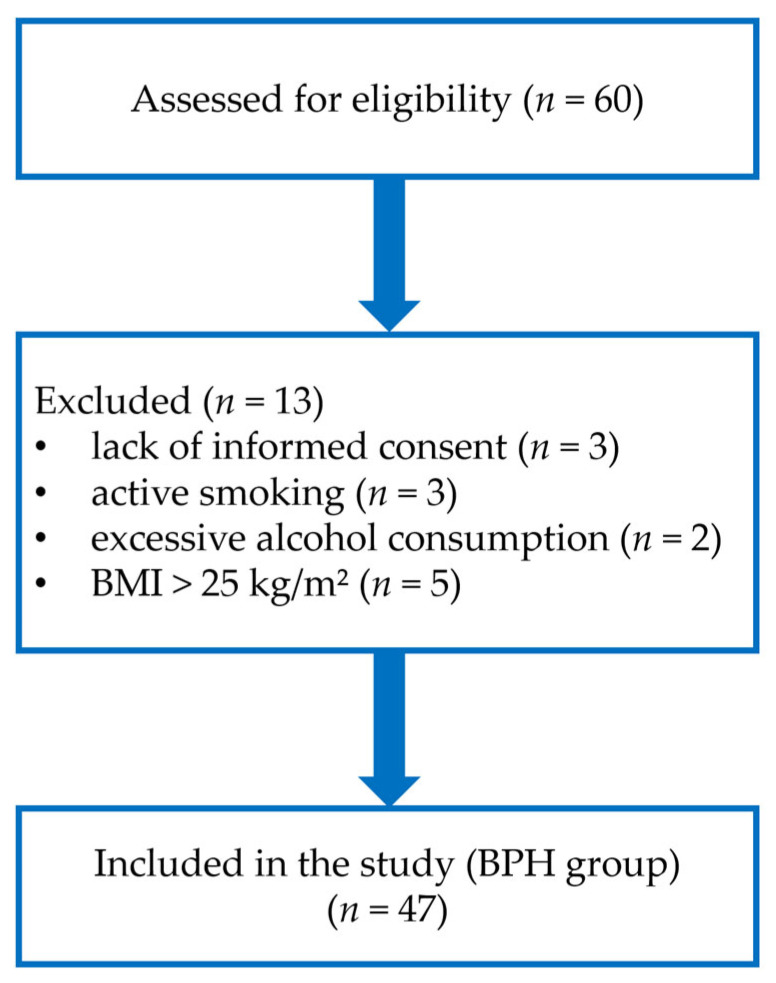
Flow chart of patient screening and exclusion in the BPH study.

A total of 60 consecutive men diagnosed with BPH and qualified for transurethral resection of the prostate (TURP) due to moderate or severe LUTS or urinary obstruction were assessed for eligibility in this single-center, cross-sectional study. Participants were recruited continuously between March and September 2022 by a specialist urologist (K.B.) at the Clinical Department of Urology and Urologic Oncology, Municipal Hospital in Rzeszów, Poland. Demographic characteristics, medical history, and relevant lifestyle information were recorded for all screened individuals. Thirteen patients were excluded due to lack of informed consent (*n* = 3), active smoking (*n* = 3), excessive alcohol consumption (*n* = 2), or body mass index (BMI) > 25 kg/m^2^ (*n* = 5). The final study group consisted of 47 patients with confirmed BPH who were included in the analysis. A detailed flow chart of patient recruitment and exclusion is presented in Figure 1.

#### 2.2.1. Inclusion Criteria

The study enrolled only patients with a first-time diagnosis of BPH confirmed by a urologist, prostate enlargement documented by transrectal ultrasound and/or digital rectal examination, clinical qualification for TURP due to moderate or severe lower urinary tract symptoms, and no history of previous pharmacological or surgical treatment for BPH.

#### 2.2.2. Exclusion Criteria

Participants were excluded if they: declined to participate, reported active smoking or chronic alcohol consumption, had a BMI > 25 kg/m^2^, had undergone chemotherapy or radiotherapy, had a history of endocrine disorders, dyslipidemia, diabetes mellitus, obesity, malignancies other than BPH, presented with evidence of an active inflammatory process, had a history of recurrent urinary tract disease or previous interventions for prostate disorders. Routine urinalysis was performed to exclude clinically significant urinary tract infections. Patients with overt urinary tract infection were not included in the study. The strict exclusion of overweight individuals may have reduced metabolic heterogeneity, potentially amplifying detectable redox differences. These criteria were applied to minimize potential confounding factors related to metabolic disturbances, chronic inflammation, or lifestyle-associated oxidative stress.

#### 2.2.3. Control Group

The control group included 40 healthy men, selected to approximate the age distribution of the BPH group. Controls were recruited from individuals attending routine health examinations at a local outpatient clinic during the same period (March–September 2022). Eligible controls: had no history of LUTS, were free from chronic diseases, were not taking any medications, vitamins, or nutritional supplements for at least 30 days prior to enrollment, reported no smoking or excessive alcohol intake, and had a BMI ≤ 25 kg/m^2^.

Socioeconomic background and general dietary habits were assessed through medical interviews to support comparability between the groups. Participants were asked to maintain their usual hydration routines and to avoid alcohol, diuretics, or supplements in the days before blood sampling.

### 2.3. Materials

All essential chemicals were sourced from Sigma-Aldrich (Poznań, Poland). Absorbance was recorded with a multimode microplate reader, Tecan Infinite 200 PRO (Tecan Group Ltd., Männedorf, Switzerland). All biochemical determinations were performed in independent repetitions, unless otherwise specified, using certified analytical-grade materials and standardized, previously validated procedures.

### 2.4. Blood Sampling

Venous blood was obtained in the morning after an overnight period without food, using the S-Monovette vacuum-assisted system (SARSTEDT AG & Co. KG, Nümbrecht, Germany). After collection, blood was placed in the designated laboratory tubes and immediately centrifuged at 1000× *g* for 10 min at 4 °C to separate the serum fraction. The serum was then divided into small aliquots and frozen at −80 °C. The interval between sampling and freezing did not exceed 30 min. During all preparation steps, samples were handled on ice and shielded from light to minimize oxidative changes. In line with recommendations for the analysis of intrinsic oxidative stress markers, no stabilizers or antioxidant additives were introduced. Serum aliquots were kept for a maximum of three months before examination. Each aliquot was thawed only once, directly before measurement, after being brought to room temperature. The applied storage conditions and timeframe were selected based on prior studies indicating that oxidative stress-related biomarkers remain stable under these parameters [25,26].

### 2.5. Blood Analysis

Complete blood counts were obtained using a hematology analyzer from Siemens Healthineers (Bavaria, Germany). Serum levels of glucose, creatinine and urea were determined following standard laboratory procedures on the Cobas c501 analyzer (Roche Diagnostics, Mannheim, Germany). Coagulation tests were performed with the ACL TOP 300 CTS system (Instrumentation Laboratory, Werfen, Barcelona, Spain). The international normalized ratio (INR) was measured using the RecombiPlasTin 2G reagent, whereas activated partial thromboplastin time (APTT) was determined with the APTT-SP kit, both supplied by Instrumentation Laboratory. Serum potassium was analyzed using a liquid ion-exchange electrode method.

### 2.6. Analytical Procedures

#### 2.6.1. Protein Determination

Total protein levels were quantified using the colorimetric Lowry method [27]. For the assay, 250 µL of the Lowry reagent was pipetted into each well of a 96-well plate, after which 50 µL of serum (previously diluted) was added. The contents were mixed and allowed to react for 10 min at room temperature. Then, 25 µL of the Folin–Ciocalteu solution was introduced, the wells were mixed again, and the plate was left to stand for a further 30 min at room temperature. The optical density was subsequently read at 750 nm using a microplate spectrophotometer.

#### 2.6.2. Thiol Group Determination

Thiol group content was measured using the Ellman reaction [28]. In brief, 20 μL of serum were mixed with 2 μL of 5,5′-dithiobis-(2-nitrobenzoic acid) (DTNB) in 100 μL of 0.1 M phosphate buffer (pH 8.0) in a 96-well microplate. The mixture was incubated at 37 °C for 1 h in the dark, and absorbance was subsequently recorded at 412 nm using a reagent blank as reference. Thiol concentrations were calculated from a glutathione calibration curve and normalized to total protein content, with results expressed as nmol/mg protein.

#### 2.6.3. Total Antioxidant Capacity (TAC)

TAC was evaluated using two complementary assays.

(1) Ferric reducing antioxidant power (FRAP) assay: TAC was determined using the FRAP method [29]. Serum samples were incubated with the FRAP working reagent for 20 min at room temperature, and absorbance was measured at 593 nm. TAC values were obtained from a Trolox calibration curve and expressed as μmol Trolox equivalents per liter (μmol TE/L).
(2) 2,2′-azino-bis(3-ethylbenzothiazoline-6-sulfonic acid) radical cation (ABTS) radical cation decolorization assay: TAC was also assessed using the ABTS^•+^ decolorization method [30]. The ABTS^•+^ working solution was adjusted to an initial absorbance of approximately 1.0 at 734 nm. After adding the samples, absorbance was recorded after 6 min, and TAC values were calculated using a Trolox standard curve, expressed as μmol TE/L.

#### 2.6.4. MDA Determination

MDA concentrations were determined using the thiobarbituric acid reactive substances assay [31]. Serum samples were incubated with thiobarbituric acid and trichloroacetic acid under acidic conditions at 100 °C for 40 min, followed by centrifugation to remove precipitated proteins. The absorbance of the resulting chromogen was measured at 535 nm, and MDA levels were calculated using an extinction coefficient of 1.56 × 10^5^ M^−1^·cm^−1^. This determination was performed in duplicate.

#### 2.6.5. Assessment of 3-NT and 4-HNE Concentrations

Serum levels of 3-NT, a marker of protein nitration, and 4-HNE, a marker of lipid peroxidation, were determined using commercially available enzyme-linked immunosorbent assay (ELISA) kits (FineTest, Wuhan Fine Biotech Co., Ltd., Wuhan, China; 3-NT: catalog no. EU2560, 4-HNE: catalog no. EU0187), in accordance with the manufacturer’s instructions. Absorbance was measured at 450 nm using a microplate reader. All samples were analyzed in duplicate.

#### 2.6.6. Amadori Products Determination

Amadori products were quantified using the nitro blue tetrazolium reduction assay. Briefly, samples were incubated at 37 °C for 2 h, after which absorbance was recorded at 525 nm [32]. Concentrations were calculated using the molar extinction coefficient of monoformazan (12,640 M^−1^·cm^−1^) [25].

#### 2.6.7. AGE-Associated Fluorescence

The estimation was performed using a spectrofluorimetric method to measure novel glucose-derived fluorescence in serum [33,34], with excitation and emission wavelengths set at 350 nm and 440 nm, respectively, measured against a reagent blank and expressed as arbitrary fluorescence units normalized to protein content.

#### 2.6.8. AOPP Determination

AOPP levels were quantified spectrophotometrically at 340 nm using a modified Witko-Sarsat method [35], with chloramine-T employed as the calibration standard. Measurements were performed in duplicate and were normalized to total serum protein content and expressed as nmol of chloramine-T equivalents per mg of protein. This determination was performed in duplicate.

#### 2.6.9. Content of Tryptophan, Dityrosine, Kynurenine and N′-Formylkynurenine

The concentrations of serum tryptophan, dityrosine, kynurenine, and N′-formylkynurenine were estimated based on their fluorescence at the wavelengths of 295/340 nm, 330/415 nm, 365/480 nm, and 325/434 nm, respectively, measured against a reagent blank and expressed as arbitrary fluorescence units normalized to protein content [36]. The kynurenine-to-tryptophan ratio was calculated by dividing the fluorescence-based kynurenine value by the corresponding tryptophan value for each sample.

### 2.7. Statistical Analysis

Statistical analyses were performed using STATISTICA software (version 13.3; StatSoft Inc., Tulsa, OK, USA). The distribution of continuous variables was assessed using the Shapiro–Wilk test. As most parameters deviated from normality, data are presented as medians with an interquartile range (IQR). Variables with normal distribution are reported as mean ± standard deviation (SD).

Between-group comparisons were performed using the Mann–Whitney U test with continuity correction for non-normally distributed variables. For normally distributed variables, comparisons between two independent groups were conducted using Student’s *t*-test or Welch’s *t*-test, depending on variance homogeneity assessed by Levene’s test. All statistical tests were two-tailed.

To control for multiple comparisons across oxidative stress biomarkers, false discovery rate (FDR) correction was applied using the Benjamini–Hochberg procedure. FDR-adjusted *q*-values < 0.05 were considered statistically significant.

Spearman’s rank correlation coefficients (ρ) were calculated to evaluate associations between redox biomarkers and selected clinical and metabolic variables (age, BMI, fasting glucose, and serum urea) in the combined cohort. FDR correction was applied across all correlation analyses.

To assess independent associations between oxidative stress biomarkers and BPH status, multivariable logistic regression models were constructed. Separate models were fitted for individual biomarkers to minimize multicollinearity among correlated redox parameters. Biomarkers with non-normal distribution were log-transformed prior to modeling. Effect estimates were standardized and expressed as odds ratios (ORs) per 1 standard deviation (SD) increase to enhance comparability across variables and reduce scale-dependent inflation of estimates. In the presence of strong between-group separation and skewed distributions, effect sizes with wide confidence intervals were interpreted cautiously.

A *p*-value < 0.05 and an FDR-adjusted *q*-value < 0.05 were considered statistically significant.

## 3. Results

Baseline clinical and laboratory characteristics of the study groups are summarized in Table 1. The analysis included 47 patients with BPH and 40 healthy controls. Patients with BPH were significantly older than controls (median [IQR]: 72.0 [64.0–74.0] vs. 64.5 [56.0–72.0] years, *p* = 0.02), reflecting the age-related nature of BPH. No significant differences were observed between the groups with respect to BMI, which remained within the normal range in both groups (*p* = 0.13). White blood cell count, coagulation parameters (prothrombin time, INR, and APTT), serum creatinine, and potassium concentrations were also comparable between the groups (all *p* > 0.05), indicating the absence of acute inflammatory processes, coagulation abnormalities, or clinically relevant renal dysfunction.

Fasting serum glucose levels were significantly higher in patients with BPH compared with healthy controls (*p* < 0.01), although none of the participants met the diagnostic criteria for diabetes mellitus. Similarly, serum urea concentrations were elevated in the BPH group relative to controls (*p* < 0.01), while creatinine levels remained within the reference range in both groups.

Overall, apart from differences in age, fasting glucose, and urea levels, the study groups were comparable with respect to key clinical and laboratory parameters relevant to oxidative stress assessment. Given the age difference between the study groups, an additional age-binned subgroup analysis was performed (Supplementary Table S1).

**Figure 2:**
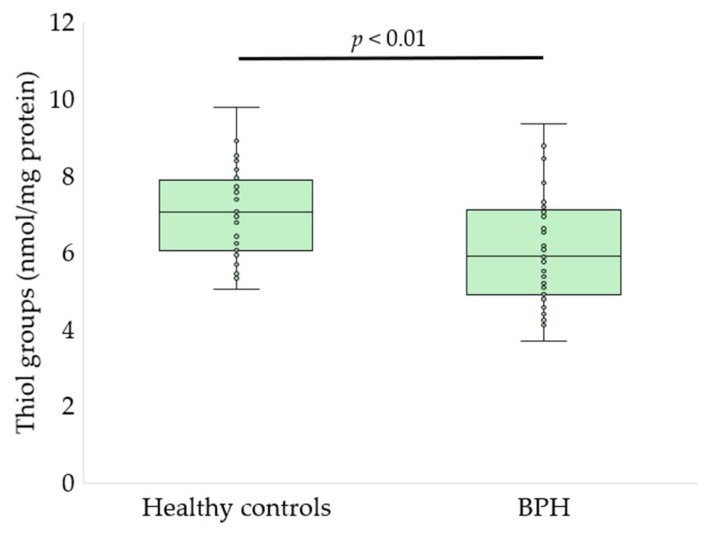
Serum thiol group content in healthy controls and patients with BPH. Data are presented as box-and-whisker plots showing the median, interquartile range, and minimum–maximum values, with individual data points overlaid (Welch’s *t*-test).

**Figure 3:**
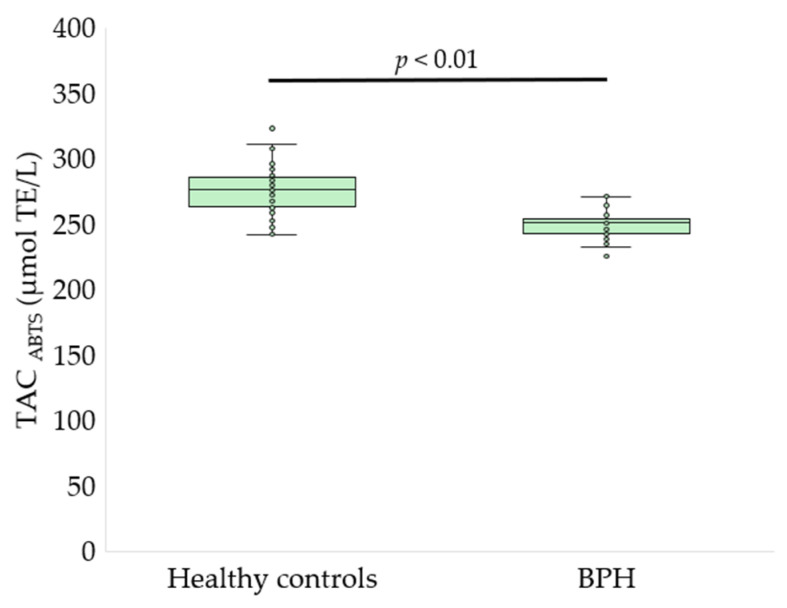
TAC assessed by the ABTS^•^ radical cation decolorization assay in healthy controls and patients with BPH. Data are presented as box-and-whisker plots showing the median, interquartile range, and minimum-maximum values; individual data points are overlaid (Welch’s *t*-test).

**Figure 4:**
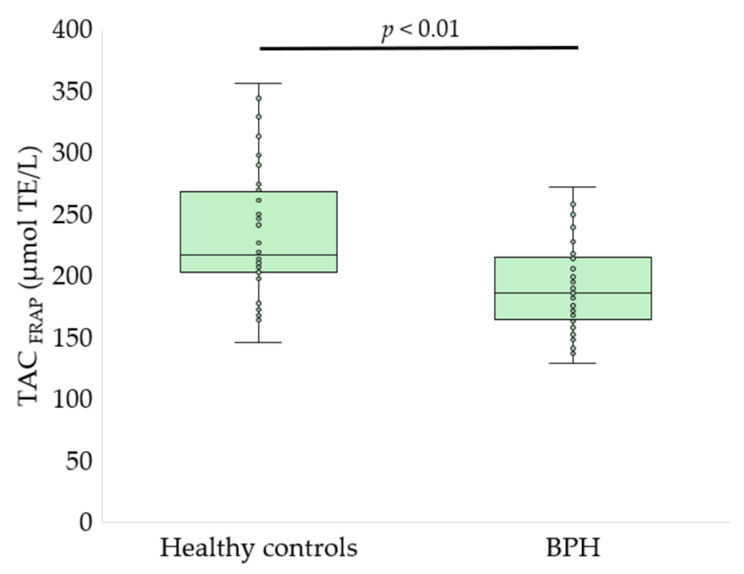
TAC assessed by the FRAP assay in healthy controls and patients with BPH. Results are presented as box-and-whisker plots showing the median, interquartile range, and minimum-maximum values, with individual data points displayed (Welch’s *t*-test).

**Figure 5:**
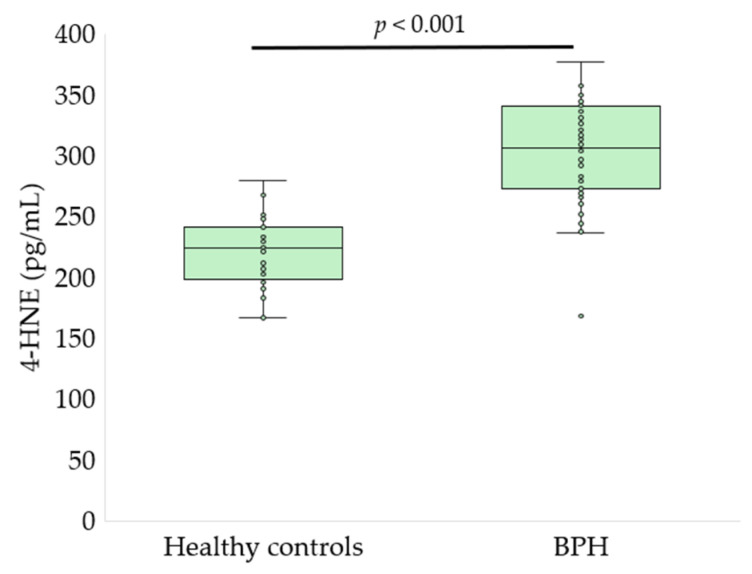
Serum 4-HNE concentrations in healthy controls and patients with BPH. Data are presented as box-and-whisker plots showing the median, interquartile range, and minimum–maximum values, with individual data points overlaid (Mann–Whitney U test).

**Figure 6:**
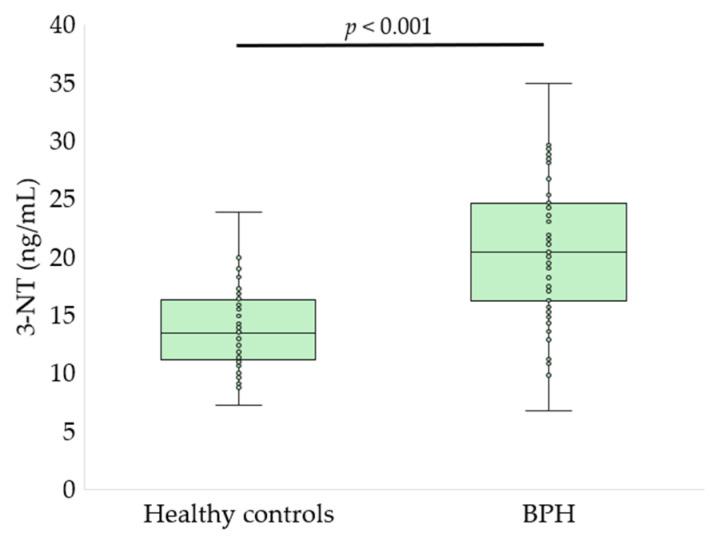
Serum 3-NT concentrations in healthy controls and patients with BPH. Data are presented as box-and-whisker plots showing the median, interquartile range, and minimum–maximum values, with individual data points overlaid (Mann–Whitney U test).

Serum oxidative stress, glycoxidation, and tryptophan metabolism markers differed significantly between patients with BPH and healthy controls (Figure 2, Figure 3, Figure 4, Figure 5 and Figure 6, Table 2).

Patients with BPH exhibited a significant reduction in serum thiol group content compared with healthy controls (6.03 ± 1.40 vs. 7.00 ± 1.16 nmol/mg protein, *p* < 0.01, *q* < 0.01; Figure 2), indicating depletion of thiol-based antioxidant defenses. Consistently, TAC was significantly lower in the BPH group when assessed using both analytical approaches. TAC measured by the ABTS^•+^ radical cation decolorization assay was reduced in patients with BPH compared with controls (250.8 ± 10.9 vs. 275.3 ± 18.6 µmol TE/L, *p* < 0.01, *q* = 0.0014; Figure 3). Similarly, TAC determined by the FRAP assay was markedly decreased in the BPH group (187.9 ± 34.2 vs. 233.8 ± 49.7 µmol TE/L, *p* < 0.01, *q* = 0.002; Figure 4).

Evaluation of lipid peroxidation and nitrosative stress markers demonstrated a significant intensification of oxidative damage in patients with BPH. Serum MDA levels were elevated in the BPH group compared with healthy controls (Table 2), indicating enhanced lipid peroxidation. In parallel, concentrations of 4-HNE, a highly reactive lipid peroxidation–derived aldehyde, were significantly higher in patients with BPH than in controls (303.2 ± 42.9 vs. 220.8 ± 27.1 pg/mL, *p* < 0.001, *q* < 0.001; Figure 5). Moreover, serum levels of 3-NT, a marker of protein nitration and nitrosative stress, were also significantly increased in the BPH group compared with healthy individuals (20.4 ± 6.0 vs. 13.9 ± 3.7 ng/mL, *p* < 0.001, *q* < 0.001; Figure 6).

Analysis of protein oxidation and glycoxidation markers revealed a modest but statistically significant increase in AGE-associated fluorescence in patients with BPH compared with healthy controls (median 3.01 vs. 2.73 a.u./mg protein, *p* = 0.031; Table 2). In contrast, dityrosine levels did not differ significantly between groups. Similarly, early glycation products (Amadori products) and AOPP showed no statistically significant differences between BPH patients and controls.

Markers of tryptophan metabolism demonstrated pronounced alterations in BPH. Kynurenine concentrations were significantly elevated in the BPH group (median 2.38 vs. 1.98 a.u./mg protein, *p* < 0.01), accompanied by a significant increase in N′-formylkynurenine (median 3.39 vs. 2.86 a.u./mg protein, *p* = 0.021). These changes were paralleled by a marked reduction in tryptophan levels in patients with BPH compared with healthy controls (88.7 ± 10.63 vs. 106.9 ± 13.87 a.u./mg protein, *p* < 0.01).

Collectively, this pattern reflects enhanced lipid peroxidation and protein nitration, depletion of systemic antioxidant capacity, increased glycoxidative stress, and activation of the tryptophan-kynurenine pathway in patients with BPH.

The kynurenine-to-tryptophan ratio, a surrogate marker of tryptophan catabolism and immunometabolic activation, was significantly elevated in patients with BPH compared with healthy controls (median: 0.027 vs. 0.019, *p* < 0.001). The effect size was large (Cohen’s d = 1.90), indicating a substantial shift in systemic tryptophan metabolism in BPH.

After FDR correction using the Benjamini–Hochberg procedure, between-group differences remained statistically significant for 11 out of 14 biomarkers. The strongest differences were observed for 4-HNE, kynurenine-tryptophan ratio, and 3-NT (all *q* < 0.001). Markers of lipid peroxidation (MDA), antioxidant depletion (thiols and TAC), and tryptophan metabolism (kynurenine, N′-formylkynurenine, tryptophan) also remained significant after adjustment. In contrast, differences in AOPP, dityrosine, and Amadori products did not withstand FDR correction.

Spearman correlation analysis performed in the entire cohort revealed that selected oxidative stress and tryptophan metabolism markers were significantly associated with metabolic parameters (Table 3). After FDR correction, the kynurenine-to-tryptophan ratio showed a strong positive correlation with fasting glucose (ρ = 0.448, *q* < 0.001) and serum urea (ρ = 0.427, *q* < 0.001). Similarly, 4-HNE was positively associated with glucose (ρ = 0.481, *q* < 0.001) and urea (ρ = 0.411, *q* < 0.001). MDA also correlated with glucose (ρ = 0.455, *q* < 0.001), whereas 3-NT demonstrated a moderate association with glucose (ρ = 0.292, *q* = 0.028).

Among antioxidant parameters, thiol groups were inversely correlated with age (ρ = −0.338, *q* = 0.007), indicating an age-related decline in antioxidant capacity. No significant associations between BMI and oxidative or kynurenine pathway markers were observed after FDR correction.

These findings indicate that while selected redox markers are partially influenced by metabolic parameters, the observed activation of the kynurenine pathway cannot be attributed solely to aging or BMI.

In multivariable logistic regression models adjusted for age, BMI, fasting glucose, and serum urea, several biomarkers remained independently associated with BPH status (Table 4). After FDR correction across models, higher levels of 3-NT, 4-HNE, MDA, AGE-associated fluorescence, kynurenine, N′-formylkynurenine, and an increased kynurenine-to-tryptophan ratio were independently linked to BPH, whereas antioxidant parameters (thiol groups, TAC) and tryptophan were inversely associated with BPH (all *q* < 0.05). In contrast, Amadori products, dityrosine, and AOPP were not independently associated with BPH after adjustment. Although several biomarkers demonstrated high odds ratios, the wide confidence intervals observed for selected parameters (e.g., 4-HNE) likely reflect limited sample size and strong between-group separation rather than true effect magnitude. Therefore, effect sizes should be interpreted with caution.

## 4. Discussion

The present study provides comprehensive evidence that BPH is associated with a distinct systemic oxidative stress profile characterized by impaired antioxidant defense, enhanced protein glycoxidation, and pronounced alterations in tryptophan metabolism. Taken together, these findings support the concept that oxidative stress is not merely a local phenomenon restricted to prostatic tissue but rather a systemic process accompanying the progression of BPH.

One of the key observations of the present study is the significant reduction in serum thiol group content in patients with BPH. Thiol-containing compounds constitute a major component of the circulating antioxidant defense system and play a critical role in redox buffering and protection against protein oxidation. Their depletion in BPH indicates an increased oxidative burden and reduced capacity to neutralize ROS [37]. Our findings are consistent with observations reported by Koike et al., who demonstrated significantly lower circulating protein thiol levels in patients with BPH compared with healthy controls (333.4 ± 47.8 µM vs. 372.8 ± 47.0 µM, respectively) [38]. In line with these data, studies evaluating thiol/disulfide homeostasis have shown that BPH is associated with significant alterations in native and total thiol levels as well as thiol/disulfide ratios, occurring independently of albumin and total protein concentrations [39,40]. Importantly, these observations are further supported by a recent systematic review and meta-analysis demonstrating significantly reduced thiol levels in patients with BPH compared with healthy controls, with BPH occupying an intermediate redox-risk position between prostate cancer patients and healthy individuals [41]. Collectively, this body of evidence reinforces the concept that attenuation of thiol-dependent antioxidant defenses represents a consistent and systemic feature of BPH rather than a phenomenon restricted to malignant prostate disease.

Consistently, TAC was significantly reduced in patients with BPH when assessed using two complementary analytical approaches, namely the ABTS^•^ radical cation decolorization assay and the FRAP assay. The concordant decrease in TAC measured by both methods strengthens the reliability of this finding and suggests a broad impairment of systemic antioxidant potential rather than selective depletion of individual antioxidants. Consistently, plasma total equivalent antioxidant capacity was also significantly reduced in a cohort of 15 patients affected by BPH compared with 15 healthy controls, further supporting impaired systemic antioxidant defenses in BPH [12]. Our findings are also consistent with recent comprehensive reviews indicating that total antioxidant status is reduced in patients with BPH compared with healthy controls, reflecting impairment of non-enzymatic antioxidant defenses, including thiol-dependent systems [42]. Together, these observations support diminished systemic antioxidant capacity as a fundamental feature of BPH-associated redox imbalance.

The concurrent elevation of MDA, 4-HNE, and 3-NT observed in the present study provides integrated evidence for enhanced lipid peroxidation and nitrosative stress in BPH. Increased serum MDA concentrations in BPH have been documented in earlier studies, indicating that lipid peroxidation is a consistent feature of oxidative stress associated with benign prostatic enlargement [43,44,45].

While MDA reflects end-stage lipid peroxidation, 4-HNE represents a biologically active aldehydic mediator capable of propagating oxidative and inflammatory signaling through protein adduct formation [46]. Previous studies have demonstrated that 4-HNE is closely linked to oxidative stress-related alterations within prostatic tissue. Immunohistochemical analyses revealed low 4-HNE expression in BPH contrasted by markedly increased accumulation in prostate cancer, particularly in poorly differentiated tumors, suggesting progressive impairment of aldehyde detoxification mechanisms during malignant transformation [47]. These findings indicate that tissue-level 4-HNE accumulation may reflect local redox dysregulation associated with cancer progression. In contrast, the present study demonstrates significantly elevated circulating 4-HNE levels in patients with BPH, suggesting that systemic lipid peroxidation may precede or occur independently of overt tissue accumulation and malignant transformation.

In parallel, increased 3-NT levels indicate peroxynitrite-driven protein nitration, linking reactive nitrogen species to oxidative damage [48]. Previous studies have demonstrated increased expression of nitrosative stress markers, including 3-NT, predominantly at the tissue level in prostate cancer compared with BPH [16]. Immunohistochemical analyses revealed higher 3-NT immunoreactivity in prostate cancer biopsies, supporting the role of protein nitration in malignant transformation of the prostate. In contrast, the present study demonstrates significantly elevated circulating 3-NT levels in patients with BPH, suggesting that systemic nitrosative stress may already be present in benign prostate pathology, preceding or occurring independently of overt malignant tissue changes.

Together, these markers suggest that BPH is characterized not only by oxidative injury, but also by sustained redox signaling driven by lipid- and nitrogen-derived reactive species.

In parallel with impaired antioxidant defenses, markers of protein glycoxidation revealed selective alterations. While early glycation products, reflected by Amadori compounds, did not differ significantly between groups, AGE-associated fluorescence was significantly higher in patients with BPH. This pattern suggests that BPH may be associated with enhanced progression from early glycation intermediates to more stable, biologically active AGE structures. AGEs are known to promote oxidative stress and inflammation through receptor-mediated mechanisms, including activation of the receptor for AGEs, which may further amplify redox imbalance and inflammatory signaling within the prostate and systemically [18]. Beyond their role as biomarkers, AGEs are increasingly recognized as biologically active mediators capable of modulating the tissue microenvironment [49]. Experimental studies in prostate cancer models have demonstrated that AGEs can promote stromal activation and epithelial cell migration through receptor for AGEs-dependent mechanisms [50]. Although these observations were made in malignant settings, they suggest that AGE accumulation may contribute to redox-sensitive stromal-epithelial interactions also in benign prostate disorders, supporting the relevance of AGE-related pathways in prostate pathophysiology. Alterations in protein glycosylation have also been reported in BPH. Structural heterogeneity of urinary prostate-specific antigen, reflected by distinct lectin-binding profiles, has been demonstrated in BPH compared with prostate cancer, indicating disease-specific modifications of oligosaccharide chains [51,52,53]. Although these changes reflect enzymatic glycosylation rather than non-enzymatic glycoxidation, they support the concept that protein carbohydrate modifications represent an integral feature of prostate pathology and may interact with redox-related processes.

Notably, markers of advanced protein oxidation such as AOPP and dityrosine did not differ significantly between groups in the present study. This observation may indicate that, at the systemic level, glycoxidative modifications and redox imbalance precede the overt accumulation of irreversible protein oxidation products in BPH. Alternatively, it may reflect differences in sensitivity and specificity among oxidative stress biomarkers, highlighting the importance of multi-marker approaches when characterizing redox status in complex chronic conditions. In contrast, Koike et al. [38] reported significantly elevated circulating levels of AOPP in patients with BPH compared with healthy controls (171.1 ± 100.2 vs. 119.0 ± 54.8 µmol/L of chloramine-T equivalents), suggesting that systemic accumulation of AOPP may depend on disease stage, patient selection, and the presence of metabolic or inflammatory comorbidities.

A particularly novel and clinically relevant finding of this study is the pronounced dysregulation of the tryptophan-kynurenine pathway in BPH. Patients with BPH exhibited significantly elevated serum levels of kynurenine and N′-formylkynurenine, accompanied by a marked reduction in tryptophan concentration. Activation of the tryptophan–kynurenine pathway is commonly linked to oxidative stress, immune activation, and chronic inflammation, as oxidative degradation of tryptophan can be driven by both reactive oxygen species and inflammatory enzymes. The observed shift toward increased kynurenine metabolites suggests enhanced oxidative and inflammatory metabolism of tryptophan in BPH, a mechanism that has been extensively described in malignant and inflammatory diseases but remains largely unexplored in benign prostatic disorders [54].

Importantly, alterations in the tryptophan-kynurenine axis may have functional consequences beyond serving as biomarkers of oxidative stress. Kynurenine metabolites are known to exert immunomodulatory effects and influence cellular proliferation, apoptosis, and vascular function [55]. Thus, activation of this pathway may contribute not only to systemic redox imbalance but also to local prostatic changes associated with tissue remodeling and disease progression in BPH. Importantly, multivariable logistic regression analysis demonstrated that several redox biomarkers remained independently associated with BPH after adjustment for age, BMI, fasting glucose, and serum urea. This indicates that the observed oxidative and kynurenine pathway alterations cannot be attributed solely to aging or mild metabolic differences between groups. In particular, 3-NT, 4-HNE, MDA, kynurenine metabolites, and the kynurenine-to-tryptophan ratio remained independently associated with BPH after multivariable adjustment. However, confidence intervals were wide for some biomarkers, indicating potential variability of effect size estimates. These findings support the concept that systemic redox imbalance represents an intrinsic biological feature of BPH rather than a secondary epiphenomenon of age-related metabolic changes.

Evidence for the involvement of oxidative stress in BPH is also provided by experimental studies using animal models. In dogs with spontaneous BPH, significantly reduced TAC accompanied by increased levels of protein oxidation markers, including dityrosine and N′-formylkynurenine, has been demonstrated in prostatic fluid and spermatozoa [56]. Interestingly, no significant differences in thiol group content were observed in this model, suggesting that the expression of thiol-dependent redox imbalance may vary depending on the biological compartment, species, and stage of the disease. Nevertheless, these findings support the concept that oxidative stress-related protein modifications represent a conserved feature of BPH pathophysiology across species.

Population-based evidence also supports a link between redox status and BPH. An analysis of NHANES data demonstrated a significant, non-linear association between the oxidative balance score (OBS) and BPH prevalence, suggesting that systemic redox-related factors are involved in BPH pathophysiology. However, as the OBS is derived from dietary and lifestyle variables rather than direct biochemical measurements, these findings highlight the complexity of redox regulation and underscore the importance of using objective oxidative stress biomarkers to characterize redox imbalance in BPH [57].

Although patients with BPH were older, multivariable adjustment demonstrated that the majority of redox biomarkers remained independently associated with BPH status, suggesting that age alone does not explain the observed biochemical phenotype. The lack of significant differences in BMI, white blood cell count, renal function markers, and total protein concentration between groups supports the interpretation that the detected redox alterations are specifically associated with BPH rather than generalized metabolic or inflammatory comorbidities. Correlation analysis revealed consistent associations between fasting glucose levels and multiple oxidative stress markers across the entire cohort. Lipid peroxidation products (MDA and 4-HNE) and protein nitration were positively correlated with glucose concentrations, whereas TAC assessed by the ABTS assay showed a significant inverse relationship. In addition, lower tryptophan levels and a higher kynurenine-to-tryptophan ratio were associated with higher glucose levels. Importantly, these associations were observed within the non-diabetic range of fasting glucose, suggesting that even subtle variations in glycemic status may be linked to systemic redox balance. This pattern indicates a coordinated redox-metabolic interaction rather than isolated biomarker fluctuations. Mild elevations in glucose may enhance oxidative burden through increased mitochondrial ROS generation, glycoxidative processes, and activation of pro-inflammatory signaling pathways [58]. Conversely, impaired antioxidant defenses may exacerbate susceptibility to oxidative damage in the context of metabolic stress.

Collectively, the observed pattern integrates antioxidant depletion, lipid peroxidation, nitrosative stress, and immunometabolic activation into a coherent systemic oxidative phenotype associated with BPH. This multidimensional redox signature may reflect persistent low-grade inflammatory activation and age-related metabolic stress contributing to prostatic remodeling.

Although we assessed only serum biomarkers, previous studies support the presence of oxidative damage also at the level of prostatic tissue in BPH. Oxidative DNA damage in transition-zone tissue has been shown to increase in BPH and to correlate with disease severity and prostate weight [59]. Earlier studies also linked oxidative DNA base lesions with altered antioxidant enzyme activity in BPH tissue [60]. Moreover, recent clinical work demonstrated that urinary inflammatory and oxidative stress biomarkers are associated with symptom severity and treatment response in BPH [23]. Nevertheless, direct correlations between serum oxidative stress markers and intraprostatic redox status remain insufficiently established; therefore, our findings should be interpreted as evidence of systemic oxidative imbalance associated with BPH rather than a direct measure of local tissue oxidative stress.

Several limitations of the present study should be acknowledged. The cross-sectional design precludes causal inference, and the lack of direct tissue-level oxidative stress assessment limits conclusions regarding local prostatic redox processes. In addition, inflammatory mediators were not directly measured, which would further clarify the relationship between oxidative stress and immune activation in BPH. Furthermore, complete and standardized data on serum PSA levels and total prostate volume were not available for all participants. In particular, the control group did not undergo detailed urological assessment, and these variables were not collected in a uniform manner across the study population. As a result, we were unable to include these clinically relevant parameters in the comparative baseline analysis. Another limitation is the lack of complete and standardized data on total prostate volume, which prevented us from analyzing potential correlations between prostate size and systemic oxidative stress markers. Moreover, due to the strict exclusion criteria applied to minimize potential confounding factors, the findings should be interpreted as applicable primarily to the selected study population meeting these criteria, which may limit the generalizability of the results to the broader BPH population. The very high odds ratio observed for 4-HNE likely reflects pronounced between-group separation in this relatively small cohort rather than the exact biological magnitude of the effect. Accordingly, the regression results should be viewed as hypothesis-supporting rather than providing definitive estimates of clinical risk.

Nevertheless, the relatively well-characterized study population and the use of multiple complementary oxidative stress markers represent important strengths of this work. From a translational perspective, the multidimensional redox signature observed in BPH may support the development of composite biomarker panels rather than reliance on single oxidative markers. Future studies integrating oxidative stress markers with clinical severity scores and imaging parameters may clarify their potential diagnostic or stratification value.

## 5. Conclusions

In conclusion, the present findings demonstrate that BPH is associated with systemic oxidative stress characterized by depletion of antioxidant defenses, enhanced glycoxidation, and activation of the tryptophan-kynurenine pathway. Collectively, these results highlight oxidative stress as a central biological component of BPH and identify tryptophan metabolism disturbances as a novel feature of this condition, warranting further investigation as potential biomarkers or therapeutic targets in benign prostatic disease.

## Figures and Tables

**Table 1:** Baseline clinical and laboratory characteristics of the study groups.

		Healthy Controls	BPH	*p*
*n*		40	47	
Age (years)	median (IQR)	64.5 (56.0–72.0)	72.0 (64.0–74.0)	0.02 ^1^
BMI (kg/m^2^)	median (IQR)	23.3 (22.2–24.3)	23.7 (23.3–24.4)	0.13 ^1^
WBC (10^3^/µL)	median (IQR)	6.7 (6.1–8.1)	7.0 (5.5–8.2)	0.74 ^1^
Prothrombin time (s)	median (IQR)	11.9 (11.5–13.0)	11.9 (11.6–12.6)	0.59 ^1^
Prothrombin time (%)	median (IQR)	93.5 (85.5–101.0)	96.0 (87.0–101.0)	0.46 ^1^
INR	median (IQR)	1.0 (1.0–1.1)	1.0 (1.0–1.1)	0.65 ^1^
APTT (s)	median (IQR)	28.9 (26.6–30.9)	29.3 (27.9–32.0)	0.25 ^1^
Creatinine (mg/dL)	median (IQR)	0.93 (0.82–1.13)	0.97 (0.84–1.05)	0.43 ^1^
Glucose (mg/dL)	median (IQR)	92.0 (85.0–97.5)	106.0 (98.0–112.0)	<0.01 ^1^
Protein (mg/mL)	median (IQR)	73.9 (69.9–77.2)	74.2 (68.5–77.2)	0.88 ^1^
Urea (mg/dL)	median (IQR)	30.0 (25.5–34.0)	39.0 (30.0–45.0)	<0.01 ^1^
K^+^ (mmol/L)	mean ± SD	4.35 ± 0.28	4.48 ± 0.48	0.11 ^2^

^1^ Mann–Whitney U Test (with continuity correction). ^2^ Welch’s *t*-test (unequal variances).

**Table 2:** Comparison of oxidative stress, glycoxidation, and tryptophan metabolism markers in healthy controls and patients with BPH. Data are presented as medians (interquartile range) for non-normally distributed variables and as mean ± SD for normally distributed variables. Between-group comparisons were performed using the Mann–Whitney U test with continuity correction for non-normally distributed variables and Welch’s or Student’s *t*-test for normally distributed variables, as appropriate.

		Healthy Controls	BPH	*p*	*q*
*n*		40	47		
MDA (μmol/L)	median (IQR)	3.39 (3.24–3.92)	4.28 (4.04–4.59)	<0.01 ^1^	<0.01
Amadori products (nmol/mg protein)	median (IQR)	1718 (1570–1834)	1637 (1534–1837)	0.50 ^1^	0.50
AGE-associated fluorescence (a.u./mg protein)	median (IQR)	2.73 (2.20–3.19)	3.01 (2.67–3.24)	0.03 ^1^	0.039
AOPP (nmol/mg protein)	median (IQR)	115 (93.8–142.0)	132 (104.3–144.8)	0.14 ^1^	0.16
Dityrosine (a.u./mg protein)	median (IQR)	1.34 (1.06–1.62)	1.44 (1.27–1.55)	0.30	0.325
Kynurenine (a.u./mg protein)	median (IQR)	1.98 (1.63–2.67)	2.38 (2.19–2.60)	<0.01 ^1^	<0.01
N′-formylkynurenine (a.u./mg protein)	median (IQR)	2.86 (2.36–3.76)	3.39 (2.96–3.74)	0.02 ^1^	0.03
Tryptophan (a.u./mg protein)	mean ± SD	106.9 ± 13.87	88.7 ± 10.63	<0.01 ^2^	<0.01

^1^ Mann–Whitney U Test (with continuity correction). ^2^ Welch’s *t*-test (unequal variances).

**Table 3:** Spearman rank correlation coefficients (ρ) between redox biomarkers, tryptophan–kynurenine pathway metabolites, and age, BMI, fasting glucose, and serum urea in the combined cohort (FDR-adjusted).

Biomarker	Age ρ	Age *p*	Age *q*	BMI ρ	BMI *p*	BMI *q*	Glucose ρ	Glucose *p*	Glucose *q*	Urea ρ	Urea *p*	Urea *q*
Thiol group	−0.338	0.001	0.007 *	−0.245	0.022	0.082	0.025	0.818	0.889	−0.201	0.061	0.156
TAC (ABTS)	−0.095	0.381	0.520	−0.134	0.217	0.347	−0.487	<0.001	<0.001 *	−0.228	0.034	0.110
TAC (FRAP)	−0.152	0.160	0.300	−0.062	0.567	0.705	−0.213	0.047	0.132	−0.184	0.088	0.197
MDA	0.216	0.045	0.132	0.219	0.0413	0.128	0.455	<0.001	0.0002 *	0.192	0.074	0.173
4-HNE	0.142	0.189	0.320	0.170	0.115	0.229	0.481	<0.001	<0.001 *	0.411	<0.001	0.001 *
3-NT	0.076	0.482	0.614	0.265	0.013	0.055	0.292	0.0061	0.028 *	0.135	0.212	0.347
Amadori products	−0.209	0.052	0.139	−0.105	0.333	0.479	0.149	0.167	0.302	−0.122	0.260	0.405
AGE-associated fluorescence	0.020	0.851	0.899	−0.078	0.473	0.614	0.003	0.980	0.980	0.357	0.001	0.004 *
AOPP	0.173	0.109	0.228	0.105	0.334	0.479	−0.029	0.791	0.885	0.172	0.110	0.229
Dityrosine	0.034	0.757	0.866	−0.035	0.747	0.866	−0.056	0.603	0.734	0.199	0.065	0.159
Kynurenine	−0.007	0.946	0.964	0.079	0.469	0.614	0.263	0.013	0.055	0.384	0.0002	0.002 *
N′-formylkynurenine	0.120	0.267	0.405	−0.024	0.826	0.889	0.013	0.906	0.940	0.380	0.0003	0.002 *
Tryptophan	−0.163	0.132	0.255	−0.100	0.357	0.500	−0.341	0.001	0.007 *	−0.234	0.0293	0.102
Kynurenine-to-tryptophan	0.046	0.672	0.801	0.143	0.186	0.320	0.448	<0.001	0.0002 *	0.427	<0.001	0.0004 *

Spearman’s rank correlation coefficients (ρ) were calculated for the entire cohort (*n* = 87). FDR correction was applied across all correlation tests using the Benjamini–Hochberg procedure. *q* < 0.05 was considered statistically significant; significant results are marked with an asterisk (*).

**Table 4:** Multivariable logistic regression for BPH status adjusted for age, BMI, fasting glucose, and urea (OR per 1 SD increase in log-transformed biomarker; *n* = 87; FDR-adjusted).

Marker	OR (95% CI)	*p*	*q* (FDR)
Thiol groups	0.18 (0.06–0.54)	0.002	0.009
TAC (ABTS)	0.13 (0.04–0.48)	0.002	0.009
TAC (FRAP)	0.12 (0.03–0.49)	0.003	0.009
MDA	3.01 (1.23–7.36)	0.016	0.025
4-HNE	452.54 (3.38–60,506.26)	0.014	0.025
3-NT	6.82 (2.23–20.87)	0.0008	0.009
Amadori products	0.57 (0.25–1.31)	0.186	0.207
AGE-associated fluorescence	2.70 (1.10–6.61)	0.03	0.041
AOPP	1.15 (0.56–2.36)	0.695	0.695
Dityrosine	1.57 (0.80–3.11)	0.192	0.207
Kynurenine	2.84 (1.26–6.38)	0.012	0.023
N′-formylkynurenine	2.57 (1.08–6.08)	0.032	0.041
Kynurenine-to-tryptophan ratio	45.77 (3.29–636.30)	0.004	0.010
Tryptophan	0.01 (0.000566–0.23)	0.003	0.009

## Data Availability

The data supporting the findings of this study are available from the corresponding author upon reasonable request, subject to applicable ethical and privacy restrictions.

## Supplementary Materials

The following supporting information can be downloaded at: https://www.mdpi.com/article/10.3390/antiox15040488/s1, Table S1: Age-stratified comparison of biochemical variables in healthy controls and patients with BPH.
