# Supplementary material for: Systemic Oxidative and Nitrosative Stress in Benign Prostatic Hyperplasia
Source: Antioxidants (Basel). 2026 Apr 14;15(4):488. doi: 10.3390/antiox15040488 (PMC13113065; doi:10.3390/antiox15040488)
Supplement: Supplementary file 1 [file antioxidants-15-00488-s001.zip › antioxidants-4237431-supplementary.pdf]

Supplementary Table S1. Age-stratified comparison of biochemical variables in healthy controls and patients with BPH

Variable	Healthy controls 56–63 years (n = 9)	Healthy controls 64–72 years (n = 11)	BPH 64–72 years (n = 16)	BPH 73–74 years (n = 9)	*p* (Healthy controls 64–72 vs BPH 64–72)
Glucose (mg/dL)	90.00 (85.00-93.00)	92.00 (83.00-97.00)	107.00 (102.75-112.50)	101.00 (94.00-111.00)	0.0002
Urea	31.00 (23.00-33.00)	29.00 (25.50-35.00)	37.00 (25.50-45.50)	42.00 (39.00-43.00)	0.1580
Thiol group (nmol/mg protein)	6.94 (6.10-7.94)	6.14 (5.71-7.12)	6.22 (4.91-7.13)	5.93 (5.51-6.53)	0.5704
TAC_{ABTS} (μmol TE/L)	262.84 (254.34-292.03)	277.11 (260.35-286.26)	247.13 (241.78-253.17)	251.56 (249.41-253.62)	0.0015
TAC_{FRAP} (μmol TE/L)	241.63 (197.79-274.33)	215.43 (205.28-270.64)	180.13 (164.37-197.52)	195.60 (164.23-217.61)	0.0028
4-HNE (pg/mL)	209.19 (202.74-232.56)	241.39 (217.34-242.28)	318.93 (286.67-338.48)	303.74 (282.65-341.06)	0.0001
3-NT (ng/mL)	11.51 (11.18-14.93)	14.26 (13.09-15.95)	19.75 (16.69-23.20)	15.25 (12.83-28.80)	0.0039
MDA (μmol/L)	3.40 (3.26-3.73)	3.58 (3.24-3.81)	4.26 (3.92-4.82)	4.11 (4.07-4.70)	0.0096
AGE-associated fluorescence (a.u./mg protein)	2.78 (2.23-3.79)	2.39 (2.23-2.82)	2.85 (2.67-3.16)	3.00 (2.84-3.03)	0.0983
Kynurenine (a.u./mg protein)	2.37 (1.78-2.64)	1.99 (1.62-2.12)	2.40 (2.20-2.60)	2.37 (2.13-2.46)	0.0015
N'-Formylkynurenine (a.u./mg protein)	2.90 (2.52-3.84)	2.84 (2.53-3.17)	3.31 (2.94-3.59)	3.35 (3.14-3.49)	0.0798
Tryptophan (a.u./mg protein)	104.84 (90.13-116.11)	101.26 (100.25-103.89)	87.62 (82.53-95.02)	86.49 (84.49-89.65)	0.0062

Data are presented as median (IQR). The p values represent the key age-overlapping comparison between healthy controls and patients with BPH aged 64-72 years (Mann-Whitney U test). Age bins were defined according to the reviewer's suggestion. *Note: The reviewer-defined age bins included 45/87 participants in total; therefore, this stratified analysis is exploratory and intended to support, rather than replace, the main age-adjusted analyses presented in the manuscript.*
